# EGFR, HER-2 and KRAS in Canine Gastric Epithelial Tumors: A Potential Human Model?

**DOI:** 10.1371/journal.pone.0085388

**Published:** 2014-01-15

**Authors:** Rossella Terragni, Andrea Casadei Gardini, Silvia Sabattini, Giuliano Bettini, Dino Amadori, Chiara Talamonti, Massimo Vignoli, Laura Capelli, Jimmy H. Saunders, Marianna Ricci, Paola Ulivi

**Affiliations:** 1 Veterinary Oncology Center, Sasso Marconi, Italy; 2 Medical Imaging, Faculty of Veterinary Medicine, Ghent University, Ghent, Belgium; 3 Istituto Scientifico Romagnolo per lo Studio e la Cura dei Tumori (IRST) IRCCS, Meldola, Italy; 4 Department of Veterinary Medical Sciences, University of Bologna, Bologna, Italy; 5 Petcare Veterinary Association, Marzabotto, Italy; National Cancer Institute, United States of America

## Abstract

Epidermal growth factor receptor (EGFR or HER-1) and its analog c-erbB-2 (HER-2) are protein tyrosine kinases correlated with prognosis and response to therapy in a variety of human cancers. KRAS mediates the transduction of signals between EGFR and the nucleus, and its mutation has been identified as a predictor of resistance to anti-EGFR drugs. In human oncology, the importance of the EGFR/HER-2/KRAS signalling pathway in gastric cancer is well established, and HER-2 testing is required before initiating therapy. Conversely, this pathway has never been investigated in canine gastric tumours. A total of 19 canine gastric epithelial neoplasms (5 adenomas and 14 carcinomas) were retrospectively evaluated for EGFR/HER-2 immunohistochemical expression and KRAS mutational status. Five (35.7%) carcinomas were classified as intestinal-type and 9 (64.3%) as diffuse-type. EGFR was overexpressed (≥1+) in 8 (42.1%) cases and HER-2 (3+) in 11 (57.9%) cases, regardless of tumour location or biological behaviour. The percentage of EGFR-positive tumours was significantly higher in the intestinal-type (80%) than in the diffuse-type (11.1%, p = 0.023). *KRAS* gene was wild type in 18 cases, whereas one mucinous carcinoma harboured a point mutation at codon 12 (G12R). EGFR and HER-2 may be promising prognostic and therapeutic targets in canine gastric epithelial neoplasms. The potential presence of KRAS mutation should be taken into account as a possible mechanism of drug resistance. Further studies are necessary to evaluate the role of dog as a model for human gastric cancer.

## Introduction

Gastric tumours are rare in dogs, representing less than 1% of all canine malignancies [1], [2]; 70–80% are epithelial adenocarcinomas [1]. As in humans, canine gastric carcinoma is more prevalent in males and is usually fatal [2]–[5]. Gastric adenomas are mainly incidental findings, although they may undergo malignant transformation [1].

In humans, gastric and gastro-oesophageal junction (GEJ) adenocarcinomas are a major cause of cancer morbidity and mortality worldwide [6]. Although complete surgical resection is the mainstay of treatment for non-metastatic diseases, many patients are not diagnosed until their disease is either locally advanced or metastatic and therefore unresectable [6]. The prognosis in advanced stages is very poor [7].

One of the most significant innovative targets in human cancer is the HER family. The members of this family, EGFR, c-erbB-2, c-erbB-3 and c-erbB-4 (also known as HER-1, HER-2, HER-3 and HER-4, respectively) are normally located on cell membranes and consist of an extracellular ligand-binding domain and an intracellular domain with tyrosine kinase activity [8].The EGF signalling pathway has been studied in human cancer patients with particular attention paid to EGFR, HER-2 and their activating ligands as the deregulation of these has been shown to play an important role in tumour initiation, progression and metastasis [7], [8]. Aberrant HER-2 expression or function has been implicated in gastric carcinogenesis and in other tumor types, including breast, ovarian, salivary gland, prostate and lung cancers [9]. Trastuzumab, a recombinant humanised IgG1*k* monoclonal antibody directed against the HER-2 extracellular domain, has shown a survival advantage in patients with metastatic HER-2-overexpressing gastric cancer [7]–[10], providing significant benefits in terms of response rate, median progression-free survival and overall survival [11]. Currently, trastuzumab in combination with chemotherapy is considered the standard treatment for patients with HER-2-positive advanced gastric cancer, and immunohistochemistry is the primary HER-2 testing method: a score of 3+ confirms eligibility for trastuzumab therapy, whereas a 1+ score indicates no overexpression. The interpretation of tumours scoring 2+ is open to debate, and FISH has been proposed to confirm HER-2 overexpression in such cases [7]–[12].

EGFR overexpression in primary gastric carcinomas and/or metastases has also been reported and is linked to a poor prognosis [13]–[15]. An increasing interest has been shown in developing immunohistochemistry-based screening methods to select patients who are eligible for treatment with cetuximab, an anti-EGFR monoclonal antibody that has proven effective in advanced colorectal cancer [15], [16].

Signal transduction between HER receptors and the nucleus is mediated by a small protein encoded by the *KRAS* gene [17]. In human oncology, the importance of the EGFR/HER-2/KRAS signalling pathway in gastric cancer is well established. *KRAS* mutations activate RAS proteins that continuously stimulate the signalling pathways in the absence of upstream stimulation of EGFR (constitutive activation). Consequently, tumours bearing a *KRAS* mutation are less likely to respond to anti-EGFR drugs [17].

In veterinary medicine, EGFR protein expression has been detected in numerous tumour types, including canine mammary tumours, primary brain tumours, nasal carcinomas, lung carcinomas and osteosarcomas [18]–[22]. HER-2 overexpression is reported in 19% to 35% of canine mammary neoplasms [23]–[25]. In a recent comparison study, Singer et al. studied canine EGFR and HER-2 expression and biology using mammary cancer cell lines, observing a substantial similarity between human and canine EGFR and HER-2 tumour-associated antigens. The antigens were recognised by trastuzumab and cetuximab antibodies, leading to growth inhibition in canine mammary cancer cell lines. This finding supports the development of novel targeted therapies and adjuvant strategies for the treatment of EGFR/HER-2-expressing canine cancers [26]. Conversely, the EGFR/HER-2/KRAS pathway has never been investigated in canine gastric cancer.

The aim of the present study was to evaluate EGFR and HER-2 immunohistochemical expression and *KRAS* mutational status in a series of canine gastric tumours. More in-depth knowledge of the role of these molecular pathways could provide new insights into the treatment of canine gastric tumours and into the evaluation of dogs as a comparative model for human cancers.

## Materials and Methods

### Ethics Statement

This study is a retrospective investigation carried out on archived tissue samples from dogs with gastric tumours. As the research did not influence any therapeutic decision in human subjects, approval by an Ethics Committee was not required. However, all diagnostic and therapeutic procedures were performed in accordance with the Public Health Service Policy on Humane Care and Use of Laboratory Animals.

All the examined samples were collected for diagnostic purposes as part of routine standard care. Owners gave informed consent to the use of clinical data and stored biological samples for teaching and research purposes.

### Criteria for Case Inclusion

Formalin-fixed and paraffin-embedded (FFPE) tissue samples from dogs with a histological diagnosis of benign or malignant epithelial gastric tumours were retrieved from the archives of the Department of Veterinary Medical Sciences, University of Bologna, Italy, the Veterinary Oncology Center, Sasso Marconi, Italy, and the Faculty of Veterinary Medicine, University of Ghent, Belgium. Only primary benign and malignant epithelial gastric tumours, as confirmed by clinical and/or post-mortem findings, were used. Endoscopic biopsies and surgical or post-mortem samples were also included. Reference haematoxylin-eosin stained sections were reviewed by an experienced veterinary pathologist (GB) to confirm the original diagnosis and to standardise the pathological classification according to WHO guidelines for the classification of gastrointestinal tumours of domestic animals [27]. Tumours were divided into two main histological groups for statistical purposes: intestinal-type (comprising tubular/papillary adenocarcinomas) and diffuse-type (comprising signet ring/mucinous carcinomas and anaplastic carcinomas) [28]. Only tissue sections containing more than 50% of tumour cells were included in the study. If available, tumour stage according to the TNM classification proposed by the American Joint Committee on Cancer was recorded [29]. EGFR/HER-2 expression was also investigated in 10 samples of normal canine gastric mucosa.

### EGFR and HER-2 Immunohistochemistry

EGFR and HER-2 expression was detected by immunohistochemistry (IHC) using commercial anti-human antibodies whose reactivity in canine tissues has previously been validated [19]–[30]. Four-micrometer-thick FFPE tissue sections were de-waxed and rehydrated. Endogenous peroxidase activity was blocked by incubation for 30 min with 3% hydrogen peroxide in distilled water. For EGFR, antigen retrieval was obtained by incubating sections in a 0.05% protease XIV (from Streptomyces griseus, P5147-1G, Sigma Aldrich, MO, USA) solution (pH 7.5) for 15 min at 37°C. Subsequently, sections were incubated for 60 min at 37°C in a humid chamber with the primary antibody (EGFR Ab-10, clone 111.6; mouse monoclonal; NeoMarkers, Fremont, CA, USA) diluted 1∶100 in phosphate buffered saline (PBS; pH 7.4, 0.01 M). Sections were incubated with a high sensitivity detection kit (EnVision Plus-HRP, Dako, Glostrup, Denmark), according to the manufacturer’s instructions.

For HER-2, antigen was retrieved with citrate buffer (2.1 g citric acid monohydrate/litre distilled water), pH 6.0, and heated for two 5-min periods in a microwave oven at 750 W. Sections were treated with a protein-blocking solution (Protein Block Serum-Free, Dako) for 20 min. Tissue sections were then incubated overnight at 4°C with the primary antibody (anti c-erb-B2 oncoprotein; rabbit polyclonal, Dako) diluted 1∶50 in a solution of 1% bovine serum albumin and PBS. Sites of primary antibody binding were identified using a commercial streptavidin-biotin-peroxidase kit (LSAB, Dako) and diaminobenzidine was used as chromogen (0.04% for 10 min at room temperature).

Sections were then counterstained with Papanicolaou’s hematoxylin. Sections of canine mammary carcinomas known to express the tested antigens were used as positive controls. Negative controls were obtained by substituting the primary antibody with an unrelated serum.

The immunohistochemical expression of EGFR was graded as follows: 0, no staining observed or membrane staining in <1% neoplastic cells; 1+, weak complete or incomplete membrane staining in >1% neoplastic cells; 2+, moderate complete or incomplete membrane staining in >1% neoplastic cells; 3+, strong complete or incomplete membrane staining in >1% neoplastic cells. Cases with an EGFR score ≥1+ were considered positive [31]. HER-2 was evaluated according to the criteria proposed by Hofmann et al [32] for human gastric cancer. HER-2 positivity (3+ IHC reaction) was defined as strong, complete or basolateral membranous immunoreactivity in at least 10% of tumour cells. In biopsies, a cluster (approximately five or more) of positively stained cells was considered positive [12]. EGFR and HER-2 expression was further evaluated in biopsy samples of gastric mucosa from ten dogs submitted to biopsy for tumour-unrelated causes.

### DNA Extraction and KRAS Gene Analysis

FFPE block-derived sections from gastric lesions were reviewed for quality and cellular content. DNA was extracted from 5-μM FFPE sections. Cells were lysed in 50 mM KCl, 10 mM Tris-HCl pH 8.0, 2.5 mM MgCl2 and Tween-20 0.45%, with the addition of Proteinase K at a concentration of 1.25 mg/ml, overnight at 56°C. Proteinase K was inactivated at 95°C for 10 minutes after which samples were centrifuged twice to eliminate debris. Supernatant was assessed for DNA quality and quantity by Nanodrop (Celbio, Milan, Italy) and was then submitted to PCR amplification.

Exon 2 of canine *KRAS* gene was amplified by PCR using the following primers: forward 5′-CTG CACTGAATTTTCTGAAGCA-3′ and reverse 5′ AAA ATG GGC CTG CAC AAA T′. PCR products were purified using the Minielute PCR purification kit (Qiagen, Hilden, Germany) and then submitted to sequencing using the BigDye Terminator 3.1 Reaction Cycle Sequencing kit (Applied Biosystems, Foster City, CA, USA). Sequence reactions were purified using the DyeEx 2.0 Spin kit (Qiagen) and separated by capillary electrophoresis with laser-induced fluorescence detection (3130 Genetic Analyzer, Applied Biosystems). Sequencing products were analysed in comparison with the wild type sequence of the gene.

### Statistical Analysis

The association between EGFR/HER-2 expression (positive/negative) and clinical pathological parameters (tumour stage, location, malignancy and histotype) were tested for significance with Fisher’s exact test. Significance was set at p<0.05. Tests were carried out with GraphPad Prism 5.0 (GraphPad Software, San Diego, CA, USA).

## Results

Nineteen samples were collected (7 endoscopic biopsies, 8 surgical samples and 4 post-mortem samples). There were five benign tumours (26.3%; 1 tubular adenoma, 1 papillary adenoma, and 3 tubulopapillary adenomas) and 14 gastric carcinomas (73.7%; 1 in situ carcinoma, 2 tubular adenocarcinomas, 1 papillary adenocarcinoma, 1 tubulopapillary adenocarcinoma, 8 mucinous/signet ring cell carcinomas and 1 undifferentiated carcinoma). There were 5 intestinal-type (35.7%) and 9 diffuse type carcinomas (64.3%) according to Lauren’s classification [28]. Tumours were located in the cardia (n = 1; 5.9%), gastric fundus (n = 2; 10.5%), lesser curvature (n = 6; 31.6%) and pyloric antrum (n = 8; 42.1%). In two (10.5%) cases, tumour location was unavailable. No statistical correlation was found between tumour location and histological type. Grossly, diffuse-type tumours most often appeared as a diffuse thickening of gastric mucosa (*linitis plastica*), while intestinal-type tumours frequently had the appearance of polypoid-like lesions or mucosal plaques with ulceration. Among malignant tumours, 4 (40%) were stage I, 2 (20%) were stage III and 4 (40%) were stage IV. The mean age of the affected dogs was 10.8 years for adenomas and 8.8 for carcinomas (range 3–16 years); 12 dogs were males (63.2%) and 7 were females (36.8%). There were 3 Boxers, 2 Shih-tzus, 5 crossbreeds, and 9 dogs of other breeds. Animal details are summarised in Table 1.

**Table 1:** Animal details and tumor characteristics in 19 cases of canine gastric epithelial neoplasms.

Case	Breed	Age,yrs	Sex	Tumor location	Type of sample	Histological diagnosis	TNM Stage*	EGFRscore	HER-2score	KRAS gene analysis
1	Shih-tzu	8	M	Pyloric antrum	Surgical	Tubular adenoma	N/A	0	2+	Wild type
2	Shih-tzu	10	MC	Pyloric antrum	Surgical	Papillary adenoma	N/A	3+	3+	Wild type
3	Crossbreed	7	M	Pyloric antrum	Surgical	Tubulopapillary adenoma	N/A	0	3+	Wild type
4	Crossbreed	13	M	Pyloric antrum	Surgical	Tubulopapillary adenoma	N/A	2+	3+	Wild type
5	Crossbreed	16	FS	Cardia	Endoscopic biopsy	Tubulopapillary adenoma	N/A	1+	2+	Wild type
6	Boxer	3	M	Pyloric antrum	Surgical	In situ carcinoma within tubulopapillary adenoma	TisN0M0/Stage 0	1+	3+	Wild type
7	Italian Hound	8	F	Pyloric antrum	Post mortem	Tubular adenocarcinoma	T3NxM1/Stage IV	1+	3+	Wild type
8	Chow-Chow	12	M	Lesser curvature	Post mortem	Tubular adenocarcinoma	T3N1M0/Stage IIIA	3+	3+	Wild type
9	Crossbreed	7	FS	–	Endoscopic biopsy	Papillary adenocarcinoma	T1NxM0	0	2+	Wild type
10	Italian Griffon	12	F	–	Post mortem	Tubulopapillary adenocarcinoma	T2NxM1/Stage IV	1+	3+	Wild type
11	Boxer	9	M	Gastric fundus	Post mortem	Mucinous/signet-ring cell carcinoma	T3N1M0/Stage IIIA	0	2+	Wild type
12	Boxer	10	M	Gastric fundus	Surgical	Mucinous/signet-ring cell carcinoma	T4N1MX/Stage IV	0	3+	Mut G12R
13	Pyrenean Mountain Dog	10	F	Lesser curvature	Endoscopic biopsy	Mucinous/signet-ring cell carcinoma	T1N0M0/Stage IA	0	3+	Wild type
14	German Shepherd	10	F	Pyloric antrum	Endoscopic biopsy	Mucinous/signet-ring cell carcinoma	T1N1M0/Stage IB	0	3+	Wild type
15	Dalmatian	10	F	Lesser curvature	Surgical	Mucinous/signet-ring cell carcinoma	T4N1M0/Stage IV	1+	3+	Wild type
16	Golden Retriever	8	M	Lesser curvature	Surgical	Mucinous/signet-ring cell carcinoma	T2N0M0/Stage IB	0	2+	Wild type
17	Shar Pei	6	M	Pyloric antrum	Endoscopic biopsy	Mucinous/signet-ring cell carcinoma	T1N1M0/Stage IB	0	2+	Wild type
18	Bouvier des Flandres	9	M	Lesser curvature	Endoscopic biopsy	Mucinous/signet-ring cell carcinoma	T1NxM0	0	2+	Wild type
19	Crossbreed	10	M	Lesser curvature	Endoscopic biopsy	Undifferentiated carcinoma	N/A	0	–	Wild type

N/A, not applicable;

TNM staging system for gastric carcinomas according to the classification of the American Joint Commission on Cancer^11^.

### EGFR and HER-2 Expression

**Figure 1:**
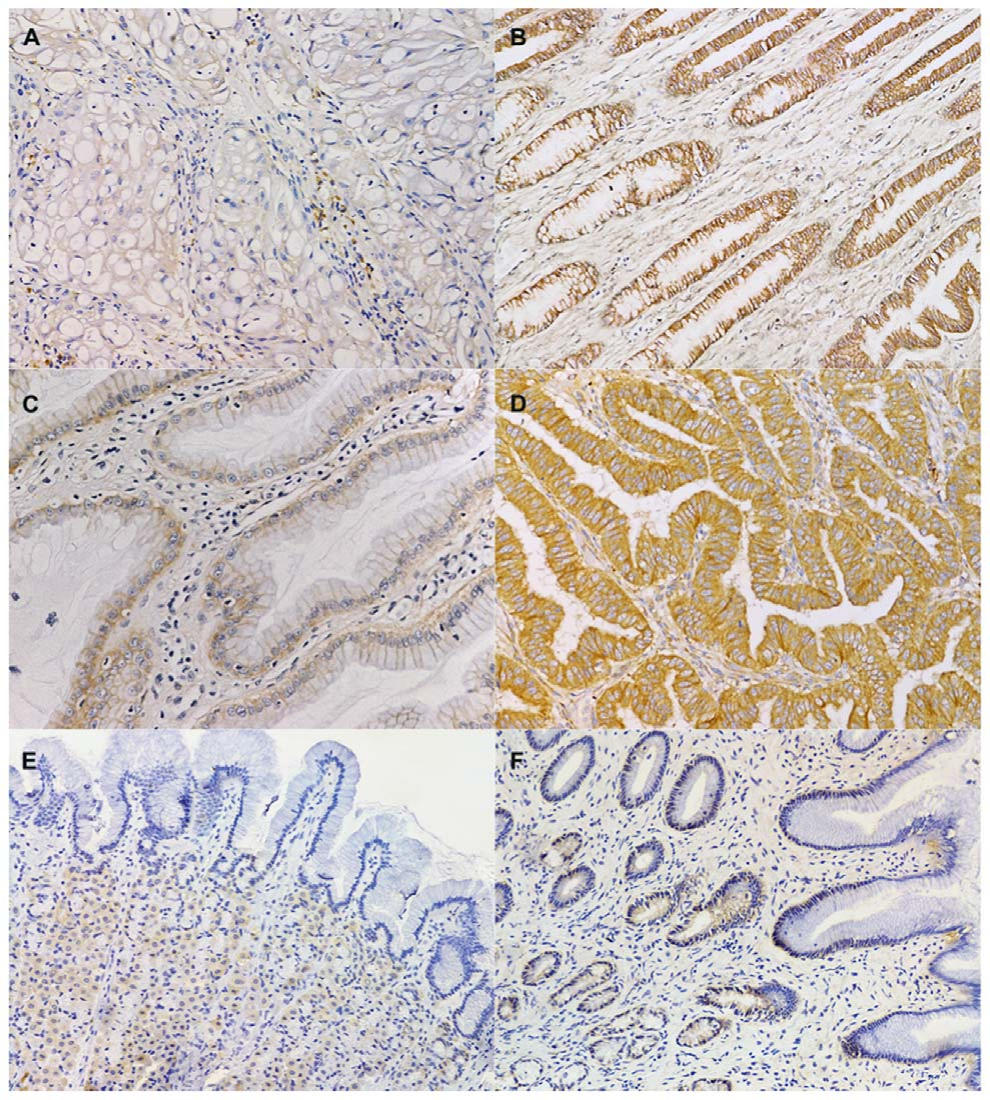
EGFR and HER-2 immunohistochemistry of canine samples. (**A**). Dog, stomach. Signet ring cell carcinoma (case No. 15). EGFR immunohistochemistry. Faint and partial membrane labelling of the neoplastic cells (1+). Haematoxylin counterstain. 200x. (**B**) Dog, stomach. Papillary adenoma (case No. 2). EGFR immunohistochemistry. Strong and complete membrane labeling of the neoplastic cells (3+). Haematoxylin counterstain. 100x. (**C**) Dog, stomach. Tubulopapillary adenoma (case No. 4). HER-2 immunohistochemistry. Moderate basolateral membrane labeling of the neoplastic cells (2+). Haematoxylin counterstain. 200x. (**D**) Dog, stomach. In situ tubulopapillary carcinoma (case No. 6). HER-2 immunohistochemistry. Strong and complete membrane labeling of the neoplastic cells (3+). Haematoxylin counterstain. 200x. (**E**) Dog, stomach, fundus. EGFR immunohistochemistry. Negative labeling of the mucosal epithelium. Faint granular cytoplasmic positivity of the parietal cells. Haematoxylin counterstain. 200x. (**F**) Dog, stomach, pyloric antrum. HER-2 immunohistochemistry. Scattered foci of faint basolateral positivity. Haematoxylin counterstain. 200x.

The EGFR protein was expressed in 8 (42.1%) out of 19 samples. Five (26.3%) cases were scored as 1+, 1 (5.3%) case was scored as 2+ and 2 (15.8%) cases were scored as 3+ (Figure 1a,b). No differences were observed in EGFR expression on the basis of tumour stage, location or malignancy. The percentage of EGFR-positive lesions was significantly higher among intestinal-type (80%) than diffuse-type (11.1%, p = 0.023) tumors (Table 2). Eleven (57.9%) tumours were considered HER-2 positive (3+). In the remaining cases (n = 7; 36.9%), HER-2 expression was classified as 2+ (Figure 1c,d). In one dog (case 19), HER-2 analysis was not possible because of tissue exhaustion. No statistically significant differences in HER-2 positivity were observed on the basis of clinical pathological parameters (Table 2). In adenomas, HER-2 staining was more intense in dysplastic areas.

**Table 2:** Association between EGFR/HER-2 status and clinical pathological parameters in 19 canine gastric epithelial neoplasms.

Parameter	EGFR ≥1+(*n* = 8)	P	HER-2 3+ (*n* = 11)	P
**Sex**		ns		ns
* Male*	4/12		6/11	
* Female*	4/7		5/7	
**Age, years**		ns		ns
* <10 years*	2/9		3/9	
* >10 years*	6/10		8/9	
**Localisation**		ns		ns
* Gastric fundus*	2/6		4/7	
* Pyloric antrum*	4/8		6/8	
**Malignancy**		ns		ns
* Adenomas*	3/5		3/5	
* Carcinomas*	5/14		8/13	
**Histotype**		0.023		ns
* Intestinal type*	4/5		4/5	
* Diffuse type*	1/9		4/8	
**Stage IV**		ns		ns
* Yes*	3/4		4/4	
* No*	1/6		3/6	

EGFR/HER-2 expression was not detected in the 10 samples of normal gastric mucosa, with the exception of scattered foci of weak basolateral positivity (Figure 1e,f).

### KRAS Gene Analysis

**Figure 2:**
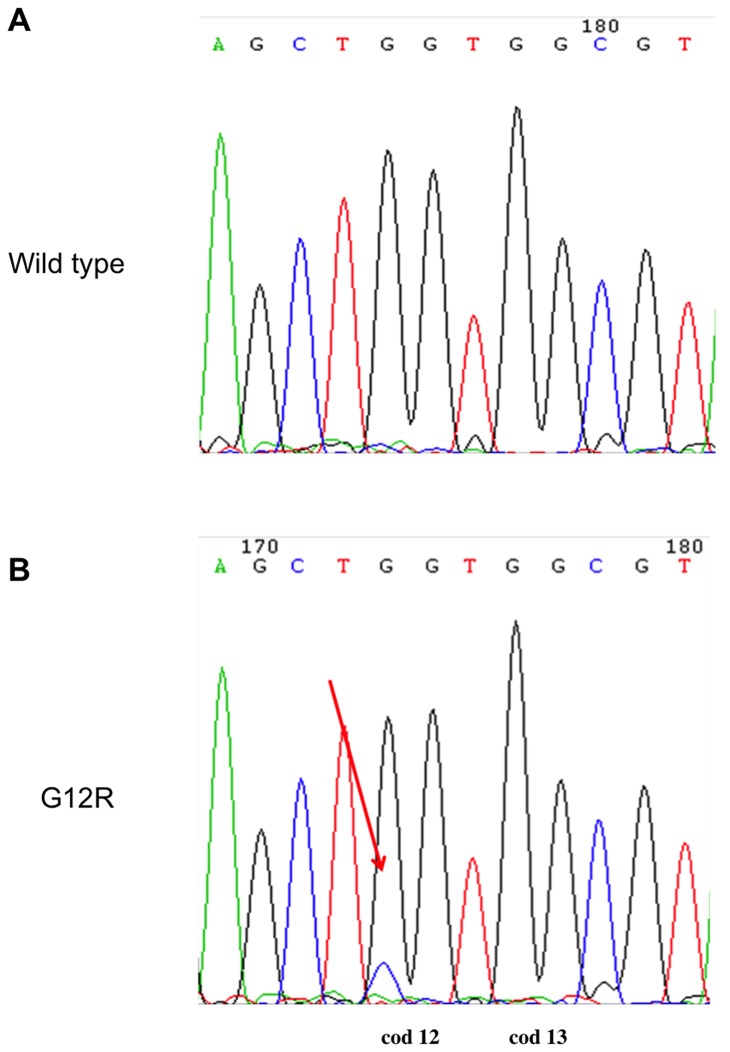
KRAS analysis. Mutation analysis performed by direct sequencing on wild type (**A**) and mutated (**B**) samples. Arrows indicate the point mutation.

All five adenomas showed a wild type *KRAS* gene status. Of the 14 carcinomas, 13 were *KRAS* wild type and one was mutated. The identified mutation was a G to C transversion at the first position of codon 12 of the gene (GGT → CGT (G12R)), which induces the substitution of a glycine with an arginine. The mutation was found in stage IV mucinous/signet-ring cell carcinoma (Figure 2).

## Discussion

HER-2 and EGFR are transmembrane tyrosine kinases that can promote tumour genesis and progression. Expression of HER-2 and EGFR appear to be closely related, and one or both proteins are frequently overexpressed in gastric epithelial cancer cells [8]. In recent years, new developments in cancer biology have led to the emergence of novel molecular-targeted therapeutics. These drugs act selectively on cancer cells at a molecular, biochemical and genetic level, specifically targeting abnormal cells, with minimal effects on the function of normal cells [8]. As in humans, the prognosis of canine gastric cancer is very poor and currently available therapeutic aids fail to significantly prolong survival. The need for new treatment options thus encourages in-depth studies on the role of these molecules as potential therapeutic targets in veterinary medicine as well. Additionally, strong similarities have been observed between human and canine gastric cancer with regard to clinical presentation and histopathological features, which indicates the dog as a potential comparative model for human gastric cancer.

Our retrospective study was conducted on 19 cases of canine gastric epithelial tumours, the small number of cases possibly constituting a limitation.Data on clinical stage were not available in six dogs (nos. 1, 2, 3, 4, 5 and 19). A 1.7∶1 male to female ratio was observed, confirming the male predisposition previously reported by other authors [2]–[5]. Additionally, our data support the preferential localisation of these tumours to the pyloric antrum and lesser curvature [33]–[35].

Overall, both receptors were expressed in a high percentage of cases (EGFR: 42%, HER-2: 61%). All HER-2 negative cases were also negative for EGFR expression, whereas a subset of EGFR-negative tumours was found among HER-2 positive tumours. Immunohistochemical expression was membranous, complete or basolateral. EGFR positivity among tumors was extremely heterogeneous, whereas we did not observe major intra-tumour differences in HER-2 expression.

In humans, HER-2 immunohistochemical expression varies according to tumour location, with a higher rate of HER-2 positivity in GEJ tumours compared to those located in the gastric body (34% vs 20%) [11].The single most important factor for the development of these tumours is the mucosal irritation caused by chronic GE reflux. In such cases squamous epithelium is eroded and replaced by columnar epithelium, either by intestinal metaplasia or by the extension of columnar epithelium from the stomach (Barrett’s oesophagus) [35], [36].

HER-2 expression in humans also differs significantly on the basis of histological subtype. Intestinal-type cancers usually exhibit higher rates of HER-2 positivity compared to diffuse-type tumors (34% vs 6%)^9^. No clear correlation has been found between EGFR expression and tumour location or histotype.

Among the cases in this study, only one tumour was located in the GEJ (HER-2, 2+); the remaining lesions were almost equally distributed between the gastric fundus and the pyloric antrum, with no significant difference in receptor expression. Conversely, a higher percentage of intestinal-type than diffuse-type carcinomas were positive for both markers. However, this difference was only statistically significant for EGFR.

In human gastric cancer, HER-2 is involved in the development of relatively early-stage carcinogenesis [12]. Additionally, although the majority of studies conducted in human medicine link the overexpression of HER-2 to adverse prognosis [12], a small number have not found any a correlation with the biological behaviour of the tumour [37]. Likewise, EGFR expression has been associated with increased tumour aggressiveness [15]. Given the retrospective nature of this study, it was not possible to trace the clinical follow-up of the majority of cases. Nevertheless, some indication about tumour biological behaviour can be inferred from IHC expression in gastric mucosa samples from controls compared to those from adenomas and carcinomas.

Neither receptor was expressed in non-neoplastic gastric mucosa, indicating their possible involvement in carcinogenesis. Additionally, in benign tumours, HER-2 was more intensely expressed in the focal areas of dysplasia, suggesting its potential relevance in premalignant lesions. However, as no appreciable differences in marker expression were observed between adenomas and carcinomas and between locally advanced and metastatic cancer, a correlation with tumour biological behaviour would seem unlikely.

In human oncology it has been observed that the KRAS gene is affected by a limited number of mutations, more than 90% involving codons 12, 13 and 61 (exons 2 and 3). These mutations are associated with the constitutive activation of the gene and are considered responsible for resistance to treatment with anti-EGFR monoclonal antibodies [38].

In gastric carcinomas, the reported frequency of KRAS point mutations is between 8% and 10% [39], [40]. Although KRAS mutations represent a prognostic factor for colorectal and lung cancer, their correlation with the biological behaviour of gastric tumours is still poorly defined [38]–[40].

The nucleotide sequence of the canine KRAS gene is very similar to that of the human one, and the resulting amino acidic sequence is identical^41^. Kraegel and coworkers analysed KRAS activation in a subset of canine non-small cell lung cancers: despite the wide disparity in the incidence of non-small cell lung cancer between dogs and humans, the frequency of KRAS point mutation was similar. Although species-specific factors may be responsible for mutations, exposure to common environmental carcinogens may account for some of the identified similarities in KRAS activation [41].

Mutation analysis of KRAS performed on our samples showed the presence of a single mutation at codon 12. This mutation is among the most frequently detected in humans and determines the substitution of a glycine (the only amino acid without a side-chain) by an amino acid with a side-chain (arginine), thus leading to a geometric alteration of the protein. This results in a lack of GTP hydrolysis which keeps KRAS in a permanently activated state [42]. The mutation was found in a mucinous carcinoma of the gastric fundus characterised by a massive infiltration of adjacent structures and regional lymph node metastases. Unfortunately, no information was available on the clinical course of the case, a 10-year-old male boxer. The potential presence of KRAS mutation in dogs should be taken into account when considering to use of targeted drugs against the HER pathway as it could represent a mechanism of resistance [43].

In conclusion, the present study revealed a high expression of EGFR and HER-2 in canine gastric epithelial tumours, which suggests a role of these receptors in carcinogenesis, especially when compared to the constant negativity of normal gastric mucosa. Additionally, there was a significantly higher percentage of EGFR-positive cases among intestinal-type carcinomas. Unlike humans, however, we did not observe a relationship between marker expression and anatomical location or the biological behaviour of tumours. Finally, a codon 12 mutation in the KRAS gene was identified, equivalent to those found in human gastric carcinomas, suggesting that this altered pathway may also exert a role in the pathogenesis of gastric cancer in dogs.

The potential relevance of these molecules as prognostic and predictive markers indicates the need for further studies on larger case series involving the use, in parallel, of *in situ* hybridisation and immunohistochemistry.

## Conclusions

The pathological and behavioural similarities between many spontaneous canine and human tumours make logical to extend investigations into molecular oncogenesis to dogs. Therapeutic targeting of EGFR and HER-2 could be a promising line of research in canine gastric cancer. Further studies are needed to evaluate the role of the dog as a model for human gastric cancer.
