# Correction: EGFR, HER-2 and KRAS in Canine Gastric Epithelial Tumors: A Potential Human Model?

**DOI:** 10.1371/journal.pone.0119048

**Published:** 2015-03-05

**Authors:** The PLOS ONE Staff

The affiliations for the 10^th^ and 11^th^ authors are incorrect. Marianna Ricci and Paola Ulivi are not affiliated with #2 but with #3 Istituto Scientifico Romagnolo per lo Studio e la Cura dei Tumori (IRST) IRCCS, Meldola, Italy.
